# Transcultural adaptation and validation of the Chinese version of the Visceral Sensitivity Index for patients with disorders of gut-brain interaction

**DOI:** 10.3389/fmed.2026.1810265

**Published:** 2026-04-29

**Authors:** Mingyu Fu, Yang Chen, Liang Gong, Zhifeng Wang, Xiucai Fang, Yanping Duan, Xiaoqing Li

**Affiliations:** 1 Department of Gastroenterology, Peking Union Medical College Hospital, Chinese Academy of Medical Sciences & Peking Union Medical College, Beijing, China; 2 Department of Psychological Medicine, Peking Union Medical College Hospital, Chinese Academy of Medical Sciences & Peking Union Medical College, Beijing, China

**Keywords:** Chinese version, disorder of gut-brain interaction, gastrointestinal-specific anxiety, transcultural adaptation, validity, Visceral Sensitivity Index

## Abstract

**Background:**

Gastrointestinal-specific anxiety (GSA) is a critical psychosomatic factor influencing symptom perception and quality of life in disorders of gut-brain interaction (DGBI). The Visceral Sensitivity Index (VSI) is a validated instrument used to assess GSA in DGBI such as irritable bowel syndrome (IBS). However, a culturally adapted and psychometrically validated Chinese version is currently unavailable. This study aimed to develop and comprehensively validate a Chinese version of the VSI (VSI-C).

**Methods:**

The VSI-C was developed through dual translation, back-translation, cultural adaptation, and pilot testing. Its content validity was assessed by an expert panel. The included DGBI patients completed the VSI-C, the Hospital Anxiety and Depression Scale (HADS), and the Patient Health Questionnaire-15 (PHQ-15). Structural validity was evaluated using exploratory factor analysis (EFA), with the factor model tested by confirmatory factor analysis (CFA) and further corroborated visually via exploratory graph analysis (EGA). Convergent validity was assessed through Pearson correlations with the HADS and PHQ-15. Internal consistency and split-half reliability were evaluated using Cronbach’s *α* and the Spearman-Brown coefficient, respectively. Floor and ceiling effects were analyzed based on score distributions. Finally, receiver operating characteristic (ROC) curve analysis was conducted to determine the optimal clinical cutoff score.

**Results:**

A total of 102 DGBI patients were included. The scale demonstrated excellent content validity (S-CVI/Ave = 0.91). EFA identified a stable four-factor structure, which was confirmed by CFA with good model fit (χ^2^/df = 1.539, RMSEA = 0.073, CFI = 0.910) and visually corroborated by EGA. The VSI-C showed significant positive correlations with HADS-A, HADS-D, and PHQ-15 scores (all *p* < 0.05), supporting convergent validity. It exhibited good internal consistency (Cronbach’s *α* = 0.861) and split-half reliability (Spearman-Brown coefficient = 0.860). No floor or ceiling effects were observed. The optimal cutoff score was 41.5, with a sensitivity of 67.3% and a specificity of 75.5%.

**Conclusion:**

The VSI-C is a reliable, valid, and clinically useful tool for assessing GSA in Chinese DGBI patients. Its implementation facilitates the systematic integration of psychological assessment into clinical management, promoting personalized care within a biopsychosocial framework.

## 1 Introduction

Disorders of gut-brain interaction (DGBI), formerly referred to as functional gastrointestinal disorders (FGIDs), are characterized by chronic or recurrent gastrointestinal symptoms without any underlying organic disease, affecting up to 40% of the general population worldwide (1). Common DGBI in clinical practice include functional dyspepsia (FD), irritable bowel syndrome (IBS), and functional constipation (FC) etc. Patients with DGBI frequently seek medical assistance due to persistent and recurrent symptoms, significantly impacting their health-related quality of life and imposing substantial healthcare burdens (2). Currently, it is widely accepted that the pathogenesis of DGBI is multifactorial (3), and the biopsychosocial model provides a framework to understand the etiology and clinical features (4). Previous research has indicated comorbid psychological disorders, particularly anxiety and depression (prevalence 30–50%), influence the symptom onset, progression and treatment response (5). Anxiety subtypes in DGBI patients include generalized anxiety disorder (GAD), social anxiety, and symptom-specific anxiety (SSA). Among them, SSA, termed gastrointestinal-specific anxiety (GSA), involves heightened concern or excessive vigilance toward gastrointestinal sensations, symptoms, or symptom-triggering scenarios, resulting in cognitive, affective, and behavioral responses (6). Different from GAD, which encompasses anxiety across multiple domains, GSA specifically focuses on gastrointestinal symptoms, manifesting as hypervigilance or attentional bias (7). Moreover, evidence suggests that GSA not only contributes to the pathophysiology and symptoms of IBS (8) but also negatively impacts the patient’s quality of life (9, 10). Therefore, early identification of GSA is crucial for effectively managing IBS and other DGBI (11–13).

Psychological questionnaires are now routinely used in clinical practice to screen for psychological disorders such as anxiety and depression, assess disease severity, and monitor treatment outcomes and quality of life. However, there is a lack of scales to assess GSA in DGBI. General anxiety measures may fail to capture the specific cognitive and behavioral aspects of GSA. Therefore, it is essential to establish a reliable and valid questionnaire to evaluate the psychological status of DGBI patients. The Visceral Sensitivity Index (VSI) was originally developed to assess the severity of GSA in IBS patients (14). The scale has been translated into Norwegian (15), Japanese (16), and Ukrainian (17) versions, and demonstrated reliability and validity in IBS populations. Recent research also validated the VSI in gastroparesis patients (18) and eating disorder (19). However, few studies (20, 21) applied the VSI to assess GSA in diarrhea-predominant IBS (IBS-D) patients, and its use in other DGBI remains limited. Therefore, this study aimed to: (1) cross-culturally adapt the VSI into a Chinese version (VSI-C); and (2) comprehensively evaluate its reliability, validity and clinical utility in a Chinese DGBI patient cohort. This work seeks to provide a needed tool for enhancing the integrated biopsychosocial management of DGBI in China.

## 2 Materials and methods

### 2.1 Participants

Patients diagnosed with DGBI, who visited the Gastroenterology Outpatient Clinic at Peking Union Medical College Hospital (PUMCH) from January 1st 2024 to April 30th 2024, were eligible for inclusion. Inclusion criteria included the following: (a) age 18–70 years; (b) confirmed DGBI diagnosis according to Rome IV criteria (FD, IBS, and FC) by two experienced gastroenterologists (4); (c) ability to complete questionnaires independently without hearing, speaking or comprehension impairments; (d) capacity to sign informed consent personally or via family members. Exclusion criteria were: (a) presence of organic gastrointestinal diseases (e.g., tumors, inflammatory bowel disease, etc) or history of gastrointestinal surgery; (b) communication difficulty or cognitive impairment preventing questionnaire completion; (c) refusal to sign the informed consent.

The sample size was determined based on the common rule of 5 to 10 participants per item for analysis (22). With 15 items in the VSI, a target of 75 to 150 participants was established.

The study was approved by the Medical Ethics Committee of PUMCH (Approval No. I-24PJ0298).

### 2.2 Measures

#### 2.2.1 Visceral Sensitivity Index

The VSI is a 15-item self-report questionnaire originally developed to assess the severity of GSA in patients with irritable bowel syndrome (14). It measures GSA across five conceptual domains: worry, fear, vigilance, sensitivity, and avoidance. Each item is rated on a 6-point Likert scale (1 = “strongly agree” to 6 = “strongly disagree”), with scores being reverse-coded from “0” to “5” (i.e., 1–6 transforms 5–0). The total scores range from 0 to 75, with higher scores indicating more severe GSA symptoms.

The Chinese version (VSI-C) was developed following Brislin’s translation model (23). This process involved: (1) independent translation by two bilingual translators (one English language specialist, one DGBI specialist), resulting in a preliminary Chinese version (Version 1.0); (2) back-translation by two other bilingual translators (one English language specialist, one medical professional) blinded to the original scale, resulting in an English version (Version 2.0); (3) cultural adaptation by experienced specialists in gastroenterology and psychiatry, offering evaluations based on their professional knowledge, clinical experience, language practices, and cultural backgrounds, which yielded Version 3.0. After conducting a pilot survey on Version 3.0, further adjustments were made based on feedback and expert recommendations, ultimately resulting in the official VSI-C (Supplementary Table 1).

#### 2.2.2 The Hospital Anxiety and Depression Scale

The Hospital Anxiety and Depression Scale (HADS) is suitable for screening anxiety and depression symptoms in comprehensive hospital settings. The scale consists of 14 items, with 7 items for anxiety (HADS-A) and 7 items for depression (HADS-D). Each item is rated from 0 to 3. Subscale scores are interpreted as: 0–7 (no symptoms), 8–10 (mild anxiety or depression), and 11–21 (moderate to severe anxiety or depression). The HADS has been extensively demonstrated good internal consistency (reliability coefficients *α* = 0.76–0.80), as well as structural validity (24, 25).

#### 2.2.3 The Patient Health Questionnaire-15 (PHQ-15)

The PHQ-15 is available to screen for somatic disorders and evaluate their severity. There are a total of 15 items, focusing on the extent of distress caused by common somatic symptoms or symptom clusters over the past 4 weeks. Symptoms are rated on a three-point scale based on severity: 0 for no distress, 1 for mild distress, and 2 for significant distress. Total scores range from 0 to 30, and are classified into four levels: no somatic symptoms (0–4 points), mild somatic symptoms (5–9 points), moderate somatic symptoms (10–14 points), and severe somatic symptoms (15–30 points) (26, 27).

### 2.3 Procedure

Patients with DGBI who met the enrollment criteria were recruited and completed the VSI-C, HADS, and PHQ-15 questionnaires with the help of evaluators. Patients were required to complete the questionnaires in a consistent setting, taking approximately 30 min.

### 2.4 Statistical analysis

Statistical calculation were conducted using IBM SPSS Statistics 26.0 (SPSS, Inc., Chicago, IL, USA), Amos version 24.0, and R software 4.3.3. Continuous variables of normal distribution were presented as means ± standard deviation (SD), and categorical data were expressed as percentages. A bilateral *p* < 0.05 was set as the standard of statistical significance.

#### 2.4.1 Content validity index (CVI)

To evaluate the content validity of the VSI-C, six experts with at least 10 years of clinical or research experience were invited to participate in the assessment. Each expert independently rated the relevance of the 15 items for their corresponding constructs using a 4-point Likert scale ranging from 1 = “not relevant” to 4 = “highly relevant.” The item-level content validity index (I-CVI) was calculated as the proportion of experts who assigned a score of 3 or 4 to each item. Items with an I-CVI ≥ 0.78 were deemed to have adequate content validity (28). Additionally, the scale-level content validity index (S-CVI/Ave) was computed as the average of all I-CVIs to reflect the overall content validity of the scale, with a threshold of ≥ 0.90 indicating excellent overall content validity (29).

#### 2.4.2 Construct validity

Exploratory factor analysis (EFA) was performed to determine the factor structure of our study. Kaiser-Meyer-Olkin (KMO) and Bartlett’s test of sphericity were used to confirm the adequacy of EFA. Principal component analysis (PCA) and orthogonal rotation variance maximum method were used to extract factors (factor loading > 0.40) (30). Based on the EFA results, confirmatory factor analysis (CFA) was used to test and confirm this hypothesized structure. We calculated the root mean square error of approximation (RMSEA), comparative fit index (CFI), and other indexes to evaluate the goodness of model fit. Adequate model fit was defined as CFI > 0.90 and RMSEA < 0.08 (31). To assess the stability of the model, a bootstrap analysis with 1,000 iterations was conducted, and bias-corrected 95% CI were calculated. We also analyzed the convergent validity of the HADS and PHQ-15 through Pearson correlation analysis.

#### 2.4.3 Exploratory graph analysis (EGA)

To supplement and visualize the factor structure obtained from EFA, we performed Exploratory Graph Analysis (EGA), a novel network psychometric approach that identifies clusters of items based on their conditional dependence relationships. All analyses were performed in R software 4.3.3 using EGAnet packages version 1.2.3 (32), which estimates a Gaussian graphical model with LASSO and then uses the Walktrap algorithm (33). For network visualization, nodes were colored by factor affiliation with soft pastel hues for clear discrimination; edges were mapped to correlation strength via two-dimensional gradient (line width and transparency), with stronger correlations represented by thicker, more opaque edges and weaker correlations by thinner, more transparent edges.

#### 2.4.4 Reliability

The reliability of the instrument was assessed in terms of internal consistency and stability. Internal consistency was evaluated using Cronbach’s alpha coefficient, and stability was measured by the split-half reliability, calculated via the Spearman-Brown formula. A coefficient value greater than 0.80 was considered to indicate good reliability (34).

#### 2.4.5 Floor and ceiling effects

To evaluate the distribution and discriminability of the total scale score, floor and ceiling effects were examined. In line with rigorous criteria for cross-cultural scale adaptation, a floor/ceiling effect was defined as ≥15% of participants scoring at the theoretical minimum or maximum, respectively (35).

#### 2.4.6 Receiver operating characteristic (ROC) curve

A ROC curve analysis was conducted to determine the cutoff value that best discriminates between participants with and without clinically elevated GSA. The reference criterion was determined by experienced gastroenterologists who integrated clinical expertise with HADS-A scores. The area under the curve (AUC), sensitivity, and specificity were calculated. The Youden index (J = sensitivity + specificity - 1) was used to determine the optimal cutoff point that maximizes both sensitivity and specificity.

## 3 Results

### 3.1 Demographics and clinical characteristics

A final sample of 102 participants was recruited, falling within the planned range, ensuring adequate statistical power for the subsequent factor analyses. The patient sample included 33 males (32.4%) and 69 females (67.6%), with a higher proportion of female patients, consistent with existing literature findings (1). The mean age was 46.5 ± 14.6 years. Most of the cohort had a higher level of education (60.8%, *n* = 62) and engaged in light physical labor (72.5%, *n* = 74). Among the participants, the diagnostic distribution was as follows: 44.1% (*n* = 45) with FD, 24.5% (*n* = 25) with FC, 13.7% (*n* = 14) with IBS, and 17.7% (*n* = 18) with overlapping DGBI. The mean score on VSI-C was 21.10 ± 12.33. The mean scores for the HADS-A and HADS-D subscales were 7.36 ± 4.32 and 6.52 ± 4.09, respectively. 49 patients (48.1%) scored above the normal threshold (> 7) on HADS-A, and 45 patients (44.1%) on HADS-D. The mean score on the PHQ-15 was 11.33 ± 5.37, with 88 patients (86.3%) scoring above the normal threshold (> 4) (Table 1).

**Table 1:** Demographics and clinical characteristics of the study participants (*N* = 102).

Items	*n* (%)
Gender	
Male	33 (32.4)
Female	69 (67.6)
Ethnics	
Han	95 (93.1)
Others	7 (6.9)
Education level	
Illiteracy	4 (3.9)
Primary school	4 (3.9)
Secondary school	32 (31.4)
Higher education	62 (60.8)
Marital status	
Single	19 (18.6)
Married	75 (73.5)
Separated	8 (7.8)
Physical labor	
Light	74 (72.5)
Moderate	22 (21.6)
Heavy	6 (5.9)
DGBI type	
FD	45 (44.1)
FC	25 (24.5)
IBS	14 (13.7)
Overlap	18 (17.6)
HADS-A	
Normal (0–7)	53 (52.0)
Mild (8–10)	21 (20.6)
Moderate to severe (11–21)	28 (27.5)
HADS-D	
Normal (0–7)	57 (55.9)
Mild (8–10)	30 (29.4)
Moderate to severe (11–21)	15 (14.7)
PHQ-15	
Normal (0–4)	14 (13.7)
Mild (5–9)	26 (25.5)
Moderate to severe (10–30)	62 (60.8)

DGBI, disorders of gut-brain interaction; FD, functional dyspepsia; FC, functional constipation; IBS, irritable bowel syndrome; HADS-A, The Hospital Anxiety and Depression Scale-Anxiety; HADS-D, The Hospital Anxiety and Depression Scale-Depression; PHQ-15, The Patient Health Questionnaire-15.

### 3.2 Content validity

Content validity assessment results indicated that the VSI-C exhibited excellent content validity. Specifically, I-CVI ranged from 0.83 to 1.00, with all items exceeding the threshold of ≥ 0.78. Additionally, S-CVI/Ave was 0.91, which surpassed the critical value of ≥ 0.90 for excellent overall content validity.

### 3.3 Construct validity

The KMO value was 0.914, and Bartlett’s test of sphericity was significant (χ^2^ = 723.248, df = 105, *p* < 0.001), confirming suitability for EFA. We used PCA to extract four factors from the 15 items based on the criterion of eigenvalues greater than 1. These four factors accounted for 60.126%. The factor loadings for all relevant items within each factor exceeded 0.4 (Table 2). Factor 1 (“Fear”) comprised VSI items 4, 8, 11, 13, 14, 15 (loadings: 0.553–0.799). Factor 2 (“Sensitivity”) comprised VSI items 6, 7, 12 (loadings: 0.697–0.828). Factor 3 (“Worry”) comprised items 1, 3, 10 (loadings: 0.444–0.814). Factor 4 (“Vigilance”) composed items 2, 5, 9 (loadings: 0.496–0.843). CFA indicated that the model fit was satisfactory, χ^2^/df = 1.539, RMSEA = 0.073, CFI = 0.910 (Table 3). Bootstrap analysis (1,000 iterations) confirmed the robustness of the model, with bias-corrected 95% CI for all items not containing 0 (*p* < 0.05 for all) (details in Supplementary Table 2).

**Table 2:** Factor loadings of exploratory factor analysis for the VSI-C.

Items	Factor 1	Factor 2	Factor 3	Factor 4
Q4: I have a difficult time enjoying myself because I cannot get my mind off of discomfort in my belly.	0.553			
Q8: As soon as I feel abdominal discomfort I begin to worry and feel anxious.	0.636			
Q11: I often feel discomfort in my belly could be a sign of a serious illness.	0.799			
Q13: When I feel discomfort in my belly, it frightens me.	0.724			
Q14: In stressful situations, my belly bothers me a lot.	0.609			
Q15: I constantly think about what is happening inside my belly.	0.697			
Q6: Because of fear of developing abdominal discomfort, I seldom try new foods:		0.697		
Q7: No matter what I eat, I will probably feel uncom-fortable.		0.828		
Q12: As soon as I awake, I worry that I will have discom-fort in my belly during the day.		0.773		
Q1: I worry that whenever I eat during the day, bloating and distension in my belly will get worse.			0.593	
Q3: I often worry about problems in my belly.			0.814	
Q10: I am constantly aware of the feelings I have in my belly.			0.444	
Q2: I get anxious when I go to a new restaurant.				0.496
Q5: I often fear that I will not be able to have a normal bowel movement.				0.843
Q9: When I enter a place I have not been before, one of the first things I do is to look for a bathroom.				0.534

**Table 3:** The model fit index of confirmatory factor analysis of the VSI-C.

Item	χ^2^/df	RMSEA	NNFI	CFI	GFI	IFI	TLI
Indices	1.539	0.073	0.875	0.910	0.871	0.904	0.875

Chi-square = 129.285, Degrees of freedom = 84.

### 3.4 Convergent validity

The HADS and PHQ-15 served as measurement tools for analyzing their correlation coefficients with the VSI-C and its four factors. Significant positive correlations were observed between the VSI-C and each of the HADS-A, HADS-D, and PHQ-15 scales (*r* = 0.363, 0.217, and 0.287, respectively; *p* < 0.05 for all). Furthermore, both the “Sensitivity” and “Vigilance” factors of the VSI-C demonstrated significant correlations with all three scales (*p* < 0.05). The “Fear” factor correlated significantly with HADS-A and PHQ-15 (*p* < 0.01 for both). However, the “Worry” factor showed no significant correlation with any of the three scales (*p* > 0.05 for all), suggesting it may capture a distinct aspect of GSA that is not fully reflected by measures of general anxiety, depression, or somatic symptom burden (Table 4).

**Table 4:** Convergent validity between VSI-C and HADS, PHQ-15.

Scale	VSI-C	Fear	Sensitivity	Worry	Vigilance
HADS-A	0.363^**^	0.362^**^	0.246^*^	0.142	0.290^**^
HADS-D	0.217^*^	0.187	0.225^*^	0.013	0.221^*^
PHQ-15	0.287^**^	0.262^**^	0.200^*^	0.167	0.219^*^

HADS-A, The Hospital Anxiety and Depression Scale-Anxiety; HADS-D, The Hospital Anxiety and Depression Scale-Depression; PHQ-15, The Patient Health Questionnaire-15; VSI-C, Chinese version of the Visceral Sensitivity Index. *, *p* < 0.05; **, *p* < 0.01.

### 3.5 EGA network structure and factor clustering

**Figure 1:**
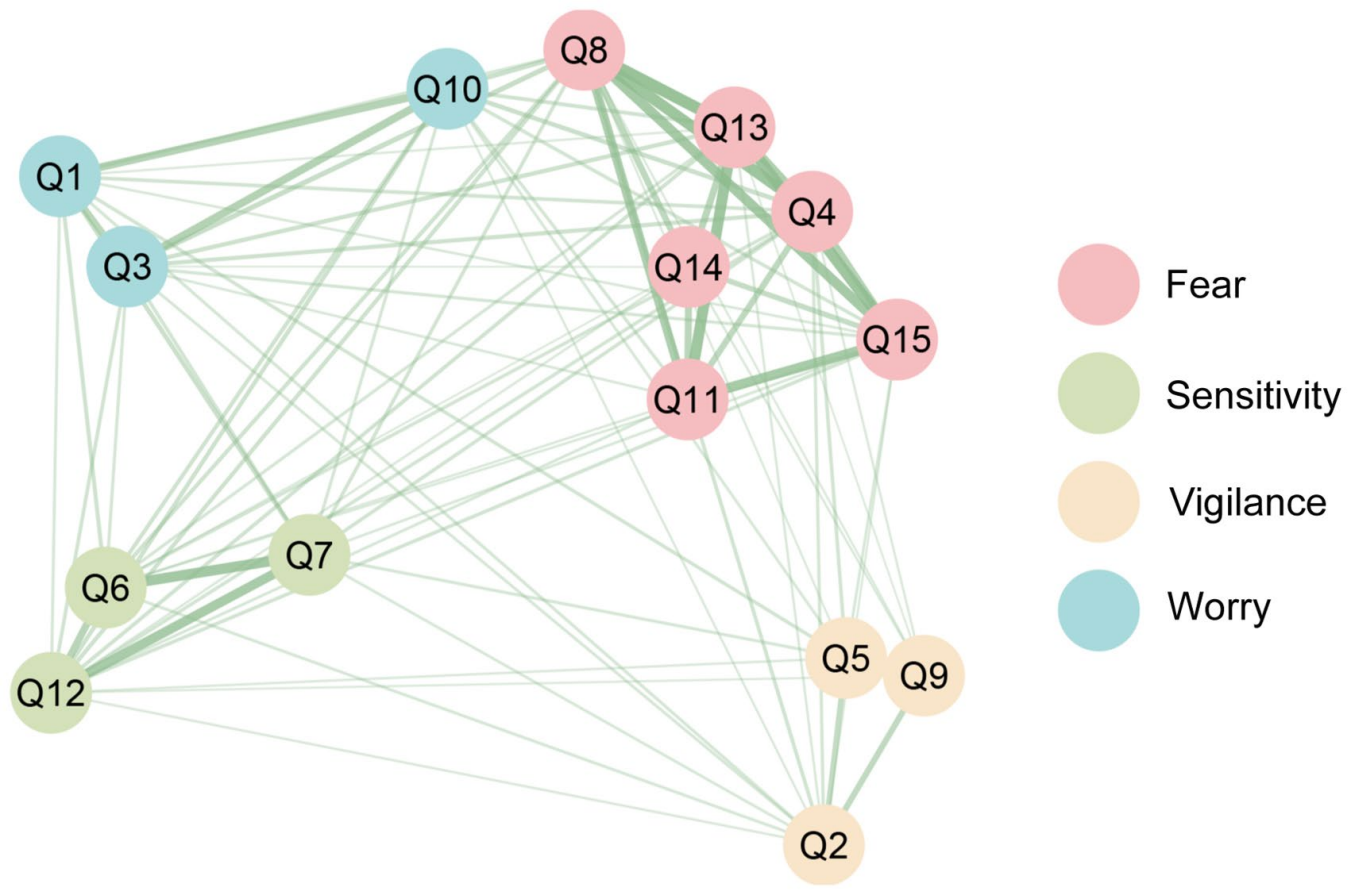
Exploratory graph analysis of the four factors of VSI-C.

EGA was employed as an additional, data-driven method to validate the factor structure identified by EFA. The resulting network model (Figure 1) clearly delineated four distinct clusters of items, with strong within-factor connections and clear separation between factors. This graphical representation visually corroborated the four-factor structure obtained through PCA. Furthermore, bootstrap resampling (1,000 iterations) demonstrated excellent stability of this model (SD = 0.04, 95% CI: 3.65–4.31).

### 3.6 Reliability

The Cronbach’s *α* coefficient of the VSI-C was found to be 0.861. Furthermore, the 15 items were divided into two parts for analysis. Due to the unequal number of items in each part, the Spearman-Brown coefficient was employed to result in a final split-half coefficient value of 0.860. Both results showed high reliability in the study.

### 3.7 Floor and ceiling effects

For the summary score of the scale, the floor effect was observed in 0.00% of participants, and the ceiling effect was found in 0.98% of participants. Both proportions were less than 1%, indicating the absence of ceiling and floor effects for the summary score.

### 3.8 ROC curve

**Figure 2:**
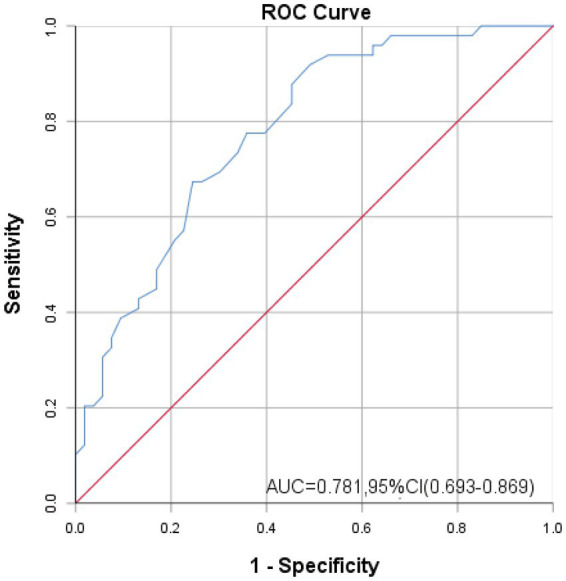
ROC curve, sensitivity and specificity of VSI-C.

The AUC was 0.781, with a sensitivity of 67.3% and a specificity of 75.5%, corresponding to a maximum Youden’s Index of 0.428. The optimal cut-off point on the ROC curve was determined to be 41.5 (Figure 2).

## 4 Discussion

This study developed and validated the first Chinese version of the Visceral Sensitivity Index (VSI-C) for patients with DGBI. The VSI-C demonstrated strong psychometric properties, including excellent content validity, good internal consistency (*α* = 0.861), and a stable four-factor structure. We established a clinically actionable cutoff score (41.5) and found significant correlations with measures of general anxiety, depression, and somatic symptom burden, supporting its convergent validity.

The rigorous translation and cultural adaptation process, underscored by excellent content validity (S-CVI/Ave = 0.91), ensured the conceptual equivalence and relevance of the VSI-C within the Chinese healthcare context. Our factor analysis yielded a stable four-factor model (“Fear,” “Sensitivity,” “Worry” and “Vigilance”), with CFI and RMSEA meeting the predefined criteria. However, the NNFI and GFI fell slightly below the conventional 0.90 threshold, may be partially attributable to the limited sample size (*N* = 102), as both indices have been shown to be sensitive to sample size, particularly in models with moderate complexity (36). The structural integrity of this four-factor model received strong, multi-method corroboration, being independently supported by both traditional confirmatory factor analysis and the novel, data-driven technique of Exploratory Graph Analysis (EGA).

Our results differed from the original US study’s one-factor model (14) and a UK study’s three-factor model (worry/awareness, fear of illness, fear of new experiences) in IBS (37). These differences may stem from our inclusion of a broader DGBI spectrum (including FD, IBS, FC). Moreover, cultural background significantly influences various psychological states, disease beliefs, symptom presentation, and coping strategies (38), potentially explaining structural variations across translated versions. This not only validates the necessity of localized adaptation but also suggests the VSI-C captures a culturally nuanced manifestation of GSA (39, 40).

Common self-reported tools for clinically assessing anxiety and depression include the HADS and the PHQ-15. Both scales provide comprehensive assessments that are easy to administer, with fine reliability and validity of Chinese versions (25, 41), as well as high sensitivity and specificity, thus serving as criterion measures. Our findings revealed a positive correlation between the VSI-C and the above scales, consistent with previous research (14, 42). However, as a particularly intriguing finding was the divergent pattern of the “Worry” factor. Unlike the other three factors and the total score, it showed no significant correlation with measures of general anxiety (HADS-A) or somatic symptom burden (PHQ-15). This suggests that, in Chinese DGBI patients, cognitive worry about gastrointestinal symptoms may represent a culturally distinct or context-specific aspect of anxiety that is not fully captured by generic psychological instruments. Regarding the three items constituting this factor (Q1, Q3, Q10), we carefully reviewed their translation. While these translations faithfully reflect the original items, the Chinese terms for “worry” carry nuanced cognitive connotations, emphasizing cognitive rumination about future consequences rather than immediate emotional distress, which may be less detectable by scales primarily assessing affective symptoms(such as the HADS-A). Of note, Item Q10 exhibited the lowest factor loading of 0.444, exceeding our predefined threshold (>0.40), and seemed to serve as a bridging node between the “Worry” and “Fear” factors in the EGA network. This bridging role may reflect the close interrelationship among attentional focus, cognitive rumination, and emotional reactivity in the experience of GSA among Chinese patients. Together, this highlights the critical value of a disease-specific tool like the VSI-C over generic measures for precise psychological phenotyping in DGBI.

The VSI-C’s significant correlations with the HADS and PHQ-15 reinforce the established gut-brain axis pathophysiology. By providing a brief, quantitative tool, this study directly addresses a gap in the assessment arsenal of frontline clinicians. Consequently, the VSI-C can be seamlessly integrated into routine gastroenterology practice, offering an objective basis to screen for GSA. A score above the identified cutoff facilitates informed clinical decisions. However, the mediating effect of GSA on the severity of IBS was controversial. A longitudinal study recruiting 276 IBS patients found that GSA could be a predictor of future deterioration in symptom severity and quality of life (43). Further research is required to investigate the effect of GSA on the severity of DGBI.

This study has limitations that chart clear directions for future research. First, the sample was recruited from a single, tertiary-care center, which may limit the generalizability of findings to community-based or primary care DGBI populations. Second, the cross-sectional design precludes causal inference, so longitudinal studies are needed to evaluate the prospective utility of the VSI-C score in predicting symptom trajectory, treatment response, and quality of life. Furthermore, future work should validate the cutoff score in an independent cohort to confirm its diagnostic accuracy.

## 5 Conclusion

In conclusion, the VSI-C is a rigorously validated tool that bridges a critical gap in DGBI care. Its implementation can facilitate early identification and targeted management of GSA, moving toward more holistic and effective patient-centered outcomes—a core goal of integrated medicine.

## Data Availability

The raw data supporting the conclusions of this article will be made available by the authors, without undue reservation.
