# Supplementary material for: Transcultural adaptation and validation of the Chinese version of the Visceral Sensitivity Index for patients with disorders of gut-brain interaction
Source: Front Med (Lausanne). 2026 Apr 29;13:1810265. doi: 10.3389/fmed.2026.1810265 (PMC13167714; doi:10.3389/fmed.2026.1810265)
Supplement: Supplementary file 1 [file Table_1.DOCX]

**Supplementary file**

**Table 1. Chinese version of the Visceral Sensitivity Index**

**内脏敏感指数量表（VSI）**

|  | **非常**  **同意** | **同意** | **有点**  **同意** | **有点不同意** | **不同意** | **非常不同意** |
| --- | --- | --- | --- | --- | --- | --- |
| 1.一天中不管什么时候进食，我都担心腹胀会加重。 | 1 | 2 | 3 | 4 | 5 | 6 |
| 2.当我去一家新餐馆时，我会感到焦虑。 | 1 | 2 | 3 | 4 | 5 | 6 |
| 3.我经常担心我的腹部问题。 | 1 | 2 | 3 | 4 | 5 | 6 |
| 4.因为我无法摆脱腹部不舒服的困扰，所以很难享受生活。 | 1 | 2 | 3 | 4 | 5 | 6 |
| 5.我经常害怕我不能正常排便。 | 1 | 2 | 3 | 4 | 5 | 6 |
| 6.因为害怕出现腹部不舒服，我很少尝试新的食物。 | 1 | 2 | 3 | 4 | 5 | 6 |
| 7.不管我吃什么，都可能感觉不舒服。 | 1 | 2 | 3 | 4 | 5 | 6 |
| 8.我一感到腹部不舒服，就开始担心和焦虑。 | 1 | 2 | 3 | 4 | 5 | 6 |
| 9.当我到一个以前没去过的地方，第一件事就是找卫生间。 | 1 | 2 | 3 | 4 | 5 | 6 |
| 10.我时常会关注腹部的感觉。 | 1 | 2 | 3 | 4 | 5 | 6 |
| 11.我经常认为腹部不舒服可能预示着某种严重的疾病。 | 1 | 2 | 3 | 4 | 5 | 6 |
| 12.我只要醒着，就担心白天腹部会不舒服。 | 1 | 2 | 3 | 4 | 5 | 6 |
| 13.当我感到腹部不舒服时，就会感觉害怕。 | 1 | 2 | 3 | 4 | 5 | 6 |
| 14.在压力大的情况下，我的腹部会更不舒服。 | 1 | 2 | 3 | 4 | 5 | 6 |
| 15.我总是在想，我的腹部发生着什么。 | 1 | 2 | 3 | 4 | 5 | 6 |

**Table 2.** **Bootstrap analysis results for factors in the four-factor model**

| **Items** | **Factor** | **Standardized loading** | **Bootstrap SE** | **Bias-corrected 95%CI** | ***P*** |
| --- | --- | --- | --- | --- | --- |
| Q4 | Fear | 0.710 | 0.075 | 0.555-0.832 | 0.001 |
| Q8 |  | 0.874 | 0.036 | 0.791-0.932 | 0.002 |
| Q11 |  | 0.664 | 0.082 | 0.468-0.798 | 0.005 |
| Q13 |  | 0.817 | 0.056 | 0.659-0.898 | 0.006 |
| Q14 |  | 0.712 | 0.063 | 0.577-0.824 | 0.002 |
| Q15 |  | 0.721 | 0.070 | 0.550-0.832 | 0.004 |
| Q6 | Sensitivity | 0.725 | 0.079 | 0.548-0.867 | 0.002 |
| Q7 |  | 0.757 | 0.070 | 0.552-0.856 | 0.006 |
| Q12 |  | 0.716 | 0.075 | 0.513-0.833 | 0.004 |
| Q1 | Worry | 0.633 | 0.086 | 0.437-0.783 | 0.003 |
| Q3 |  | 0.723 | 0.068 | 0.567-0.837 | 0.002 |
| Q10 |  | 0.460 | 0.101 | 0.225-0.626 | 0.003 |
| Q2 | Vigilance | 0.597 | 0.089 | 0.397-0.756 | 0.003 |
| Q5 |  | 0.672 | 0.089 | 0.506-0.866 | 0.001 |
| Q9 |  | 0.512 | 0.105 | 0.285-0.698 | 0.002 |

CFA, confirmatory factor analysis; SE, standard error; CI, confidence interval
